# Selective Antagonism of A1 Adenosinergic Receptors Strengthens the Neuromodulation of the Sensorimotor Network During Epidural Spinal Stimulation

**DOI:** 10.3389/fnsys.2020.00044

**Published:** 2020-07-14

**Authors:** Giuliano Taccola, Betsy Habeth Salazar, Rosamaria Apicella, Matthew Kevin Hogan, Philip John Horner, Dimitry Sayenko

**Affiliations:** ^1^Department of Neuroscience, International School for Advanced Studies (SISSA), Trieste, Italy; ^2^Department of Neurosurgery, Center for Neuroregeneration, Houston Methodist Research Institute, Houston, TX, United States

**Keywords:** motor control, spinal electrical stimulation, spinal reflexes, adenosine receptors, spinal transection, trains of pulses, terminal recordings

## Abstract

Although epidural spinal stimulation (ESS) results in promising therapeutic effects in individuals with spinal cord injury (SCI), its potential to generate functional motor recovery varies between individuals and remains largely unclear. However, both preclinical and clinical studies indicate the capacity of electrical and pharmacological interventions to synergistically increase the engagement of spinal sensorimotor networks and regain motor function after SCI. This study explored whether selective pharmacological antagonism of the adenosine A1 receptor subtype synergizes with ESS, thereby increasing motor response. We hypothesized that selective pharmacological antagonism of A1 receptors during ESS would produce facilitatory effects in spinal sensorimotor networks detected as an increased amplitude of spinally-evoked motor potentials and sustained duration of ESS induced activity. Terminal experiments were performed in adult rats using trains of stereotyped pulses at 40 Hz delivered at L5 with the local administration to the cord of 8-cyclopentyl-1,3-dipropylxanthine (DPCPX). We demonstrated that ESS combined with the blockage of A1 receptors increased the magnitude of the endogenous modulation and postponed the decay of responses that occur during ESS alone. Although DPCPX significantly increased the yield of repetitive stimulation in intact spinal cords, the effects of A1 antagonism on motor evoked responses after an acute spinal transection was not detected. These studies support the future investigation of the optimal dosage, methods of delivery, and systemic effects of the synergistic application of A1 antagonists and spinal stimulation in the intact and injured spinal cord.

## Introduction

Recent results from preclinical animal models and pilot phase clinical trials applying spinal neuromodulation have revealed that neural networks below the site of spinal cord injury (SCI) retain functional capabilities. Further, when electrically stimulated, neural networks can be reorganized to generate responses and motor activities previously thought to be permanently lost due to paralysis (Edgerton et al., 2008; Courtine et al., 2009; Fong et al., 2009; Rossignol and Frigon, 2011). Notably, epidural spinal stimulation (ESS) during activity-based rehabilitative therapy recovers previously paralyzed motor functions, improves autonomic nervous system functionality, and enhances well-being for those living with chronic paralysis due to SCI (Harkema et al., 2011; Angeli et al., 2014; Phillips and Krassioukov, 2015; Aslan et al., 2016, 2018; Grahn et al., 2017; Rejc et al., 2017; Herrity et al., 2018; Darrow et al., 2019). However, the level of functional performance regained following ESS therapy varies to a great extent, with the emergence of self-assisted stepping in a subset of trained individuals being the most advanced outcome to date (Angeli et al., 2018; Gill et al., 2018; Wagner et al., 2018). Variability in ESS efficacy likely results from unaccounted neurophysiological profiles among individuals, varying degrees of maladaptive neural plasticity, differences in training regimens, and/or high variation in spared neurologic function even within one grade of SCI severity. Given the variable effectiveness of ESS, studies that illuminate synergistic approaches (e.g., pharmacological agents) and mechanisms regulating the excitability of motor networks are needed to significantly impact the effectiveness of ESS therapy. Previous experiments in rats and cats have explored combination strategies, synergizing ESS with monoaminergic agents (e.g., clonidine, cyproheptadine, or levodopa; Courtine et al., 2009; Musienko et al., 2011) or non-competitive blockers (e.g., strychnine; de Leon et al., 1999). These works demonstrated that the spinal motor infrastructure is composed of a widely distributed and heterogeneous system of neural circuits and receptors that can generate a range of task-specific movements when recruited in different combinations (Tresch and Bizzi, 1999; Hochman et al., 2001; Courtine et al., 2009). However, researchers were unable to translate these results to the clinic, as the administration of buspirone, a serotonin agonist, produced mixed or moderate improvements (Gerasimenko et al., 2015; Gad et al., 2017; Freyvert et al., 2018). Therefore, there is a need to explore alternative pharmacological targets for more effective pharmacological neuromodulation, such as adenosine receptors (Bai et al., 2017). Adenosine is an endogenous purinergic autocoid with well-known vascular and anti-inflammatory effects (Layland et al., 2014). In the central nervous system (CNS), adenosine is synthesized by the hydrolysis of adenosine triphosphate (ATP) and then locally released by neurons and astrocytes in the synaptic cleft, where adenosine has a short half-life. Two receptors for adenosine (A1, A2) are reported in the CNS. While A1 is coupled to an inhibitory G*i* protein, A2s (A2A and A2B) are associated with a stimulating G*s* (Burnstock, 2007). The majority of A1 receptors are located presynaptically, mediating inhibition on neurotransmitter release (Fisone et al., 2004). Acting *via* A1 receptors, adenosine modulates ventral motoneurons (Witts et al., 2015) and acts on spinal motor networks as an intrinsic modulator, providing negative feedback that controls the activity generated by the spinal locomotor circuitry (Witts et al., 2011; Taccola et al., 2012; Acton and Miles, 2015). Moreover, primary afferents co-release ATP and glutamate on spinal synapses, where the resulting adenosine acts on presynaptic A1 autoreceptors to limit the flow of external input from the periphery (Burnstock and Wood, 1996). This endogenous neuromodulator inhibits afferent input from the periphery through A1 adenosine receptor subtypes, as indicated by modulation of nociceptive pathways with adenosinergic agents (Reeve and Dickenson, 1995).

An increase of endogenous adenosine during electrical stimulation has been reported arising from neuronal and glial cells (Caciagli et al., 1988; Tawfik et al., 2010). Peripheral stimulation of primary sensory afferents also releases adenosine in the spinal cord (Salter and Henry, 1987). During ESS, primary afferents are inevitably recruited and the released adenosine is a potential impediment to the yield of electrical stimulation. Indeed, A1 adenosine receptors may limit the inflow of electrical inputs, suggesting that the use of competitive antagonists for A1 adenosine receptors could facilitate the transit of electrical stimuli to neuronal networks in the spinal cord. As such, the effectiveness of electrical stimulation in recruiting a wider range of the spinal circuitry can be amplified, thus, maximizing plasticity and recovery of sensorimotor functions. Efficacy of A1 adenosine antagonists on spinal sensorimotor circuits has been studied on *in vitro* spinal cord preparations (Taccola et al., 2012), where it was demonstrated that activation of A1 adenosine receptors modulates the interneuronal networks responsible for the generation of locomotor behavior (Witts et al., 2011, 2015; Taccola et al., 2012; Acton and Miles, 2015). However, the effects of A1 adenosine antagonists on the modulation of spinally-evoked motor responses during ESS *in vivo* are currently unknown. The objective of this work is to explore whether the selective pharmacological antagonism of the subtype A1 adenosine receptors can synergize with ESS to increase spinal excitability and motor responses induced by spinal electrical stimulation. We hypothesized that the presence of A1 adenosine antagonists during spinal electrical stimulation would produce facilitatory effects in spinal sensorimotor networks, as revealed by increased amplitude of spinally-evoked motor potentials and sustained duration of the spinal electrical stimulation-induced activity. For this, we applied the A1 antagonist 8-cyclopentyl-1,3-dipropylxanthine (DPCPX) directly to the spinal cord of adult rats during supra-motor threshold ESS and assessed the neuromodulatory effects of DPCPX on spinally-evoked motor potentials. Although A2 receptors have been considered functionally marginal for the spinal sensorimotor circuits (Geiger et al., 1984), the A2 antagonist, 3,7-dimethyl-1-propargylxanthine (DMPX) was also tested in our study to explore any potential modulation of motor output evoked by ESS.

## Materials and Methods

### Experimental Design

All procedures were approved by the Institutional Animal Care and Use Committee at Houston Methodist Research Institute. Further, they were in accordance with both the guidelines of the National Institutes of Health (NIH) Guide for the care and use of laboratory animals and the European Union directive on animal experimentation (2010/63/EU). Adult Long-Evans rats (female, 300–350 g body weight; Charles River Long-Evans, Houston, TX, USA) were anesthetized by intraperitoneal (i.p.) administration of ketamine (100 mg/Kg) and xylazine (5 mg/Kg) mix and terminal electrophysiological recordings were obtained. During each experiment, toe pinches were performed periodically to assess whether the anesthetic level was maintained. If animals exhibited a reflex, a booster of ketamine was administered, as needed. Additionally, animals were kept under anesthesia over a heating pad (37°C) throughout each experiment. Finally, at the end of all experiments (2–3 h), animals were euthanized by CO_2_ followed by cervical dislocation or thoracotomy.

**Figure 1:**
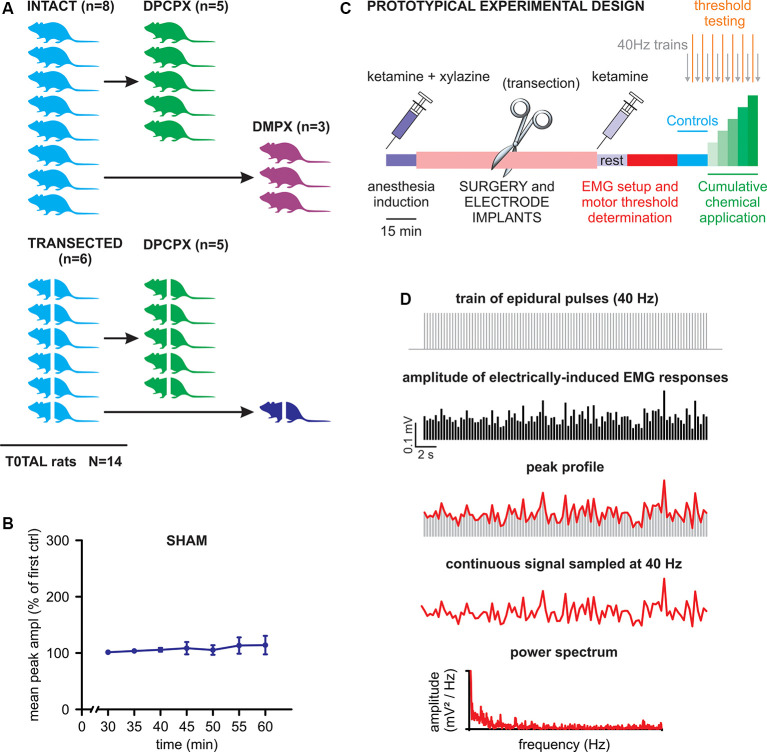
Experimental design and analysis. **(A)** The cartoon schematizes the number of animals used in the study with the corresponding experimental protocols. Animals with transected spinal cords are represented by crossed bodies. **(B)** Mean peak amplitude, expressed as a percentage of the first delivered train, is shown in the graph for all consecutive trains applied for the whole duration of a standard experiment (one train per 5 min). The values of the amplitude of electromyogram (EMG) responses are averaged from three muscles of the same animal that received consecutive stimulations without any drug application (sham). Error bars represent standard deviation (SD). **(C)** A prototypical experiment is described in its different phases (see details in the “Materials and Methods” section). **(D)** The scheme draws the procedure to quantify the rhythmic pattern modulating the amplitude of electrically-induced EMG responses during the delivery of 40 Hz trains (see “Materials and Methods” for more details).

A cartoon schematizing the experimental design of the study is provided in Figure 1A. Briefly, two main experimental groups were included: rats with intact spinal cords (*n* = 8) and rats with transected spinal cords (*n* = 6). After collecting baseline recordings to serve as internal controls for each animal, the intact cord group was further split to explore the effects of DPCPX (*n* = 5) or DMPX (*n* = 3) applications. The transected group was also further divided into those exposed to DPCPX (*n* = 5, similar to the intact-DPCPX group) and sham control (*n* = 1). For the sham control animal, trains of pulses were regularly supplied as in the treated groups, albeit without the presence of any substances. The tests demonstrated the stability of baseline responses for the entire length of a recording session after acute spinal transection (50 min). This feature is illustrated in Figure 1B, as the unchanged main amplitude of spinal reflexes, pooled from bilateral tibialis anterior (TA) and left gastrocnemius (GM) muscles, in responses to the serial delivery of 40 Hz trains every 5 min, to mimic the stimulation rate provided during real experiments [*P* = 0.490, Friedman repeated-measures analysis of variance (ANOVA) on ranks, *n* = 3].

The prototypic experimental design is schematized in Figure 1C. After a 15 min wait for the full induction of anesthesia, surgical procedures were performed (lasting approximately 1 h) for laminectomy, as well as epidural and EMG electrode implantations. Where appropriate, a complete transection of the spinal cord was performed as soon as the cord was exposed (30 min). After surgery, the second injection of ketamine was administered followed by a rest period (15 min) that was required to achieve stable conditions. Then, electrophysiological recordings began by first placing EMG electrodes and exploring the threshold intensity of epidural stimulation and then by delivering three single rectangular pulses (100 μs, duration) at increasing intensities (0.1 mA increments) with an interstimulus interval of 5 s. Motor threshold intensity was defined in each animal as the lowest intensity to elicit consistent EMG responses in a given muscle, as revealed by visual inspection of waveforms on the computer monitor. The operative suprathreshold amplitude of stimulation (range of 0.4–2 mA) was selected for each animal to evoke responses in all recorded muscles during the experiments (usually equal to 0.1 mA over the threshold value). The stability of threshold responses was verified every 5 min, 2 min after each new application. After the threshold definition, the experiments started (40–45 min) by delivering a train of 2,000 pulses at 40 Hz every 5 min. To define baseline conditions, three trains (40 Hz) were firstly supplied before the local application of drugs on the cord. Then, chemicals (DPCPX or DMPX) were applied every 5 min at increasing concentrations (1–100 μM) and the trains were repeated every 5 min before a new concentration was applied.

### Intramuscular Electromyogram (EMG) Electrode Implantation

EMG signals from bilateral TA and left GM muscles were derived from belly muscles using 13 mm paired subdermal needle electrodes (0.4 mm diameter, RLSND121-1.5, Rhythmlink Colombia, SC, USA) inserted through the shank skin. A ground electrode was placed subcutaneously on the left forearm. Before starting the experiment, proper placement of electrodes was confirmed by the motion artifact generated when tapping the muscle. EMG recordings were amplified (gain 1000, range 0.1 Hz to 1 kHz, and notched at 60 Hz) using a differential AC amplifier (DP-304A, Warner Instruments, Hamden, CT, USA) and subsequently digitalized at 40 kHz (PowerLab^®^, ADInstruments Pvt. Ltd., Bella Vista, NSW, Australia).

### Epidural Electrode Implantation and Stimulating Protocol

After a partial laminectomy between thoracic (Th)12/Th13 and the complete removal of lumbar (L)1 lamina, two multistrand, Teflon-coated stainless-steel wires (AS 632, Cooner Wire Co, Chatsworth, CA, USA) was slid through the small opening at Th12/Th13 up to L2. A small notch (0.5–1.0 mm) in each wire was deprived of insulation at L1 vertebral level (L5 spinal segment) to expose the conductor and form the electrodes, which were then placed on each side of the dorsal cord (1 mm laterally from the midline). The two wires were used as cathode and anode for bipolar stimulation. Single or repetitive current pulses (duration 100 μs) were supplied to the cord using a DS8R constant-current stimulator (Digitimer, UK). Trains of 2,000 stereotyped rectangular pulses at 40 Hz (interstimulus interval of 25 ms, the total time of the train of 50.2 s) were delivered every 5 min.

### Experimental Spinal Cord Transection and Drug Application

An acute complete transection of the cord was performed in five animals at the upper thoracic level (vertebrae Th8 to Th11) using a pair of iridectomy scissors. The resulting gap was inspected by another expert surgeon and filled by a small cotton ball for the entire duration of the experiment. DPCPX (Cat. N. 0439/100, R&D Systems Inc., Minneapolis, MN, USA) and DMPX (Cat. N. D134, Sigma-Aldrich, St. Louis, MO, USA) were locally applied to the cord by diluting each drug in 500 μl of the saline medium. In four out of 14 experiments, before adding the two pharmacological agents, the dura was opened with a longitudinal incision along the midline of the entire spinal segment L5 (leaving the dura intact under the two electrodes). Given that no difference in drug effect was found when the dura remained intact compared to when it was not intact, data were pooled from both groups. This observation confirmed previous reports on the full permeability of the spinal dura to the substances used in the current study (Nantwi and Goshgarian, 2002). The effects of pharmacologic agents were determined by comparison with internal control for each animal. Specifically, responses in DPCPX or DMPX were compared to the baseline responses collected in the same animals before the application of drugs.

### Data Analyses

Amplitude and latency (defined at the first deflection of baseline) of all EMG responses were determined using Labchart^®^ version 8 (ADInstruments, Australia) and Clampfit^®^ version 10.3 software (Molecular Devices, San Jose, CA, USA). The amplitude of all consecutive responses evoked by each train was plotted for each muscle as time-course graphs. In the animals in which simultaneous recordings were obtained from bilateral TA and left GM, all muscles showed equal responses as for latency (*P* = 0.567, one-way repeated measures ANOVA; *n* = 5) and peak to peak amplitude (*P* = 0.059, one-way repeated measures ANOVA; *n* = 5). The amplitude of suprathreshold responses in bilateral TAs and left GMs did not differ across muscle groups or among tested animals. Therefore, values were first averaged among different muscles in response to the same train, then averaged among repetitions from the same experiment, and finally pooled among different animals for statistical comparison between treatments. The sum of amplitude responses was calculated from raw values to obtain the mean cumulative amplitude for each treatment. To identify whether spontaneous variations in the peaks of responses followed a rhythmic pattern of modulation, the procedure schematized in Figure 1D was performed. In brief, the linear profile connecting the peaks of the time-course of electrically induced EMG responses was handled as a continuous waveform, with the sampling period (25 ms) equal to the interstimulus interval at the frequency of stimulation (40 Hz). The intrinsic modulatory rhythm of the amplitude of EMG reflexes was quantified in terms of power spectrum magnitude and expressed as Root Mean Square (RMS; Deumens et al., 2013), measured with Clampex 10.3^®^ (Molecular Devices Corporation, Downingtown, PA, USA). The analysis adopted a default rectangular windowing function, with data segments not overlapping, window length set at the largest value fitting within the data segments to be processed, and the first spectral bin of the periodogram excluded from RMS measurements. The magnitude of the resulting spectrum is the summed power of all rhythm frequencies. This statistical tool quantifies any increase in frequency and/or amplitude of EMG evoked-responses, expressed as a complex rhythm composed of multiple harmonics.

### Statistical Analysis

Data are indicated as mean ± standard deviation (SD) values and n refers to the number of rats used. The normality of data distribution was determined based on a Kolmogorov–Smirnov normality test. Statistical analysis was performed using SigmaStat^®^ version 3.5 software (Systat Software, San Jose, CA, USA) to compare the mean ± SD of different experimental conditions. All parametric values were analyzed using a two-tailed Student’s *t*-test (paired or unpaired) to compare two groups of data or a one-way ANOVA for more than two groups. Nonparametric comparisons were performed using the Mann–Whitney rank-sum test (unpaired) and Wilcoxon signed-rank test (paired) for two groups and Kruskal–Wallis ANOVA for more than two groups. Friedman repeated-measures ANOVA on ranks was performed for multiple comparisons. Multiple comparisons were followed by *post hoc* tests (Dunn’s Method). Results reached significance when *P* < 0.05.

## Results

### Train Pulse Delivery Elicited EMG Responses That Were Spontaneously Modulated in Amplitude

**Figure 2:**
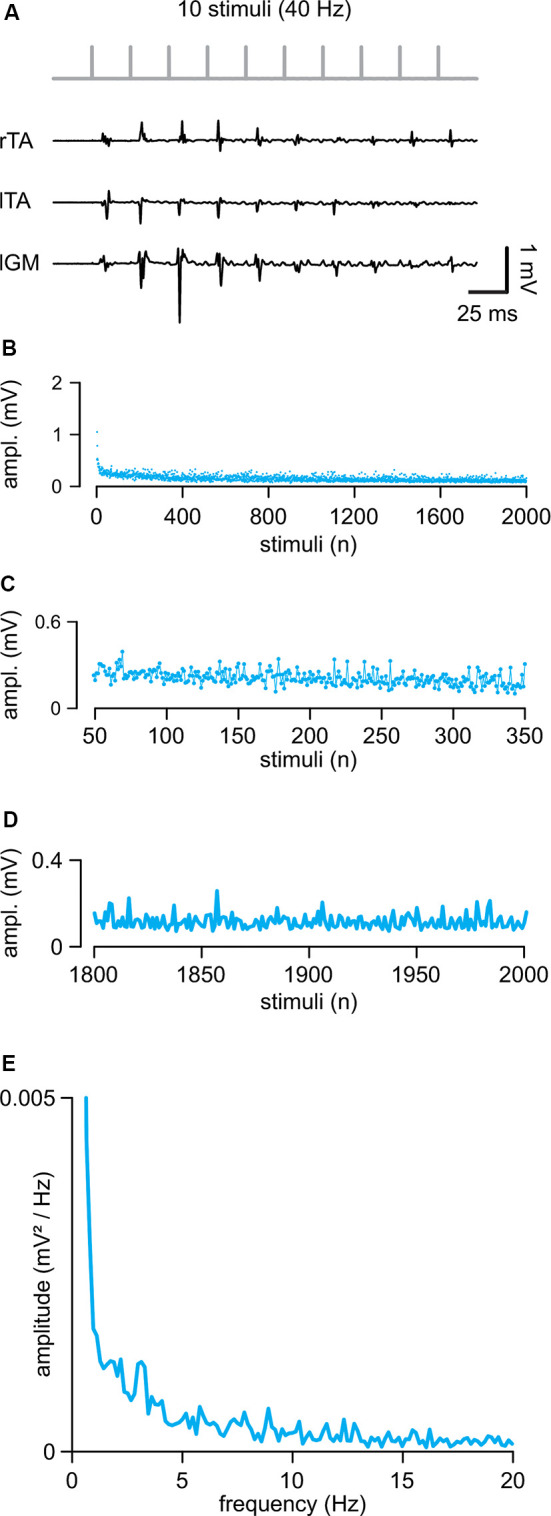
Repetitive epidural stimuli elicit responses that are rhythmically modulated in amplitude. **(A)** A train of 10 stimuli applied to the cord (L5 spinal level) at 40 Hz evokes EMG reflexes from bilateral tibialis anterior (TA) and left gastrocnemius (GM) muscles. **(B)** The Mean time-course of the amplitude of responses over the entire length of a stimulation protocol (2,000 stimuli at 40 Hz) in five animals. **(C)** Magnification of the time-course in **(B)** draws responsesto the first 300 stimuli (from the 50th to the 350th). **(D)** Magnification of the time-course in **(B)** shows the profile of peaks of the last 200 responses with their SD (from the 1,800th to the 2,000th). **(E)** The power spectrum of the mean time-course for five animals illustrates the rhythmic components of an oscillatory pattern of modulation of the peak amplitude throughout the entire stimulation protocol.

In eight intact animals, electrical stimulation of the cord (intensity = 0.71 ± 0.26 mA) evoked control suprathreshold responses with a mean latency of 5.46 ± 0.77 ms and a mean peak to peak amplitude equal to 0.57 ± 0.45 mV. In Figure 2A, sample traces from three hindlimb muscles represent the first ten responses elicited by a train of rectangular pulses (100 μs) at 40 Hz applied to the cord at the L5 spinal segment (Th13/L1 vertebrae). The amplitude of consecutive motor reflexes varied from large to minimal (up to occasional abolishment) responses. A longer train of pulses (2,000 pulses at 40 Hz) elicited responses varying in amplitude throughout the entire protocol, as summarized by the average time-course of the peak amplitudes from five experiments (Figure 2B). Motor responses consistently faded away during the first 300 pulses (7.5 s of stimulation; Figures 2B,C) with a dramatic reduction occurring after the 1,800th pulse (45 s of stimulation) under continuous stimulation at a given intensity (Figure 2D). A brief rest was sufficient to reinstate full response, as evidenced by the equal values of the cumulative amplitude of all 2,000 motor reflexes evoked by two consecutive trains spaced 5 min apart (96.64 ± 16.77%; *P* = 0.132, paired *t*-test, *t* = 1.703; *n* = 8). To identify if the spontaneous variation of responses followed a particular rhythmic pattern of modulation, the mean time-course of peaks were analyzed using power spectral analysis (Figure 2E; *n* = 5). A rhythmic component centered around 1.41 ± 0.69 Hz and with an amplitude of 0.0017 ± 0.0011 mV/Hz^2^ described the presence of a spontaneous rhythmic pattern of modulation with a power spectrum magnitude, expressed as RMS equal to 0.20 ± 0.13, *n* = 5.

These data indicate that continuous epidural stimulation with a train of pulses at 40 Hz produces EMG responses that are intrinsically modulated in amplitude by a spontaneous oscillatory rhythm of a lower frequency than the one supplied by the stimulating pattern.

### The Selective A1 Competitive Antagonist, DPCPX, Maximizes Electrical Stimulation

**Figure 3:**
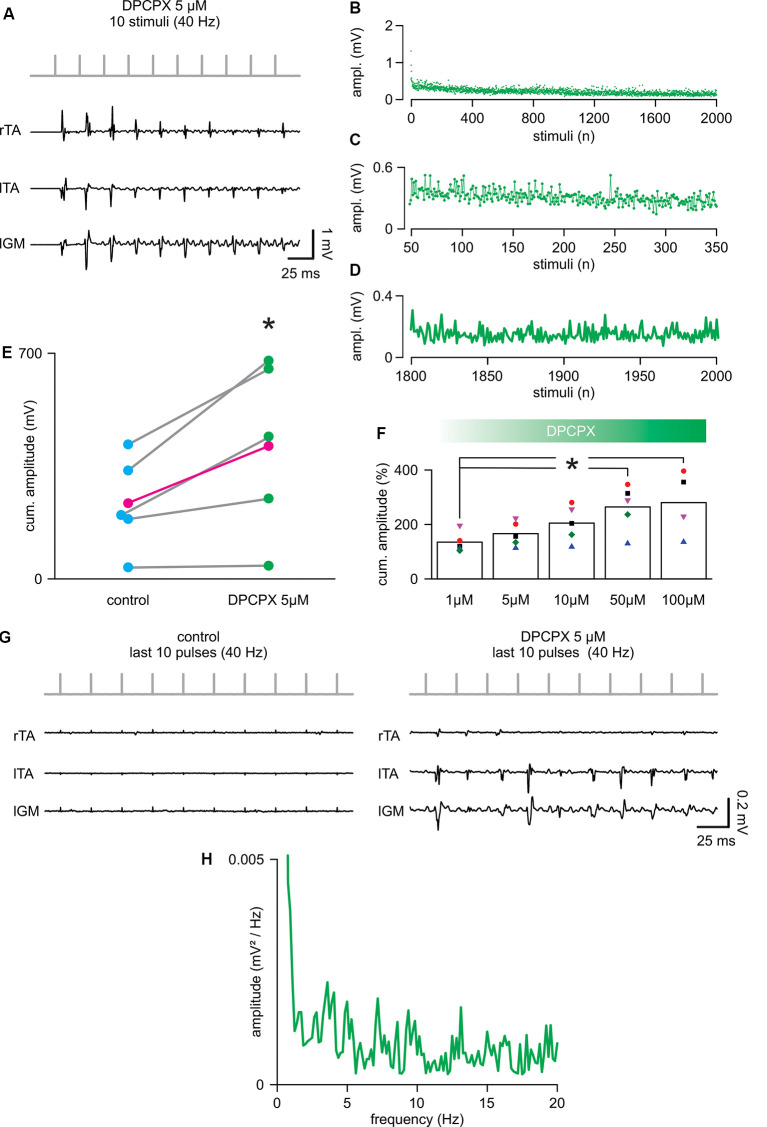
8-cyclopentyl-1,3-dipropylxanthine (DPCPX) augments reflex amplitude during repetitive stimulation. **(A)** For the same animal as in Figure 1A; DPCPX 5 μM increases the amplitude of reflexes elicited from bilateral tibialis anterior (TA) and left gastrocnemius (IGM) muscles by a train of 10 stimuli applied to the cord (L5 spinal level) at 40 Hz. **(B)** Mean time-course of the amplitude of responses over the entire length of a stimulation protocol (2,000 stimuli at 40 Hz) in combination with DPCPX (5 μM) in five animals (same animals of Figure 2). **(C)** Magnification of the time-course in **(B)** plots responsesto the first 300 stimuli (from the 50th to the 350th). **(D)** Magnification of the time-course in **(B)** shows the profile of peaks of the last 200 responses (from the 1,800th to the 2,000th). **(E)** Scatter plot of paired data reports the statistical increase of the cumulative amplitude of EMG responses elicited in five experiments by DPCPX (5 μM) during the entire electrical stimulation protocol (*P = 0.046; each pair of dots represent a single animal, while the average values are in magenta; *n* = 5). **(F)** Cumulative dose-response of the effect of DPCPX (1–100 μM) in augmenting the cumulative amplitude of EMG reflexes elicited by 2,000 stimuli at 40 Hz. Data are pooled from five animals. Data are expressed as a percentage (%) of the respective untreated controls (*P = 0.044). **(G)** Sample traces from the same animal in Figure 2A comparing responses from bilateral TAs and lGM to the last 10 stimuli of the protocol (from 1,990th to 2,000th stimuli) in control (left) and DPCPX (5 μM; right). **(H)** The power spectrum of the mean time-course for five animals (as in Figure 2) illustrates the rhythmic components of the oscillatory pattern of modulation of the peak amplitude throughout the entire stimulation protocol.

To evaluate whether the selective pharmacological blockade of the A1 adenosine receptor subtype affects spinal cord stimulation, trains of 2,000 pulses (40 Hz) were serially delivered during increasing concentrations of the selective antagonist DPCPX (1–100 μM). The application of DPCPX at any dosage did not cause spontaneous activity in the muscles. The lowest concentration tested (1 μM) did not affect the cumulative amplitude of responses (DPCPX 1 μM = 134.46 ± 37.11% of control; *P* = 0.080, paired *t*-test, *t* = −2.331; *n* = 5). Figure 3A shows, from the same animal, reported in Figure 2A; the first 10 reflexes elicited in three hindlimb muscles by a train of rectangular pulses (100 μs) at 40 Hz. The cumulative amplitude of all 2,000 motor reflexes evoked from bilateral TA and left GM by electrical stimulation is significantly increased through the administration of 5 μM of DPCPX (Figure 3E; *P* = 0.046, paired *t*-test, *t* = −2.861; *n* = 5). Figure 3F illustrates that, in five animals, increasing concentrations of DPCPX (from 1 to 100 μM) further augments the cumulative amplitude of motor responses in a dose-response manner, reaching a plateau at the highest concentrations tested (263.36 ± 84.76% and 279.16 ± 119.08% to control, for 50 μM and 100 μM, respectively; *P* = 0.044, Kruskal–Wallis one-way ANOVA on ranks followed by *post hoc* multiple comparisons vs. DPCPX 1 μM group with Dunn’s method, *n* = 5, 5, 5, 5, 4). Surprisingly, the first motor response evoked by a suprathreshold electrical stimulus applied to the spinal cord (duration = 0.1 ms; intensity = 0.78 ± 0.26 mA) was unaffected by 5 μM of DPCPX, as were the mean latency (5.44 ± 0.85 ms) and the mean peak to peak amplitude (0.50 ± 0.23 mV) of pooled data from five experiments. In the presence of DPCPX (5 μM), the average time-course of peak amplitudes showed a trend of higher responses compared to control during the first 300 pulses from the beginning of stimulation (Figures 3B,C mean amplitude = 0.24 ± 0.04 mV in control vs. 0.33 ± 0.12 mV; *P* = 0.006, paired *t*-test, *t* = −5.240; *n* = 5). Moreover, with respect to control, multiple motor responses of higher amplitude appeared for the entire duration of the stimulation protocol (Figures 3B,D). Notably, the mean amplitude of the last two hundred responses in the presence of DPCPX (5 μM) was significantly higher than the last two hundred responses in control (181.77 ± 50.52%; *P* = 0.037, paired *t*-test, *t* = −3.088; *n* = 5). Figure 3G presents sample traces of recovering reflex responses evoked by the last ten stimuli of the train from the same experimental animal as in control and after administration of DPCPX (5 μM).

The power spectrum of the spontaneous amplitude of oscillations throughout the entire protocol demonstrated the presence of a rhythmic pattern of modulation with a frequency component equal to 1.29 ± 0.51 Hz, as reported in control (Figure 3H; *P* = 0.667, Paired *t*-test, *t* = 0.464; *n* = 5) and with an unchanged amplitude of 0.0036 ± 0.0031 mV/Hz^2^ (Wilcoxon signed-rank test, *P* = 0.063; *n* = 5). However, following the administration of DPCPX (5 μM), the frequency spectrum was more populated by multiple harmonics at higher frequencies (around 3, 7, 13, and 19 Hz). Moreover, in the presence of DPCPX (5 μM), the power spectrum magnitude of rhythmic modulation significantly increased, as expressed by RMS equal to 0.32 ± 0.21 (*P* = 0.043, Paired *t*-test, *t* = 2.935; *n* = 5).

Overall, blockage of the adenosine A1 receptor subtype in combination with continuous electrical stimulation of the spinal cord increased the magnitude of the endogenous modulation pattern and postponed the decay of responses that, in fact, periodically appeared higher during the entire protocol.

In contrast, the selective A2 antagonist, DMPX, did not affect the amplitude of spinally-evoked EMG responses at any of the concentrations tested (from 1 to 100 μM; *P* = 0.995, Kruskal–Wallis one-way ANOVA on Ranks; *n* = 3).

### An Acute Transection Reduced the Amplitude of EMG Responses to Trains of Consecutive Pulses

**Figure 4:**
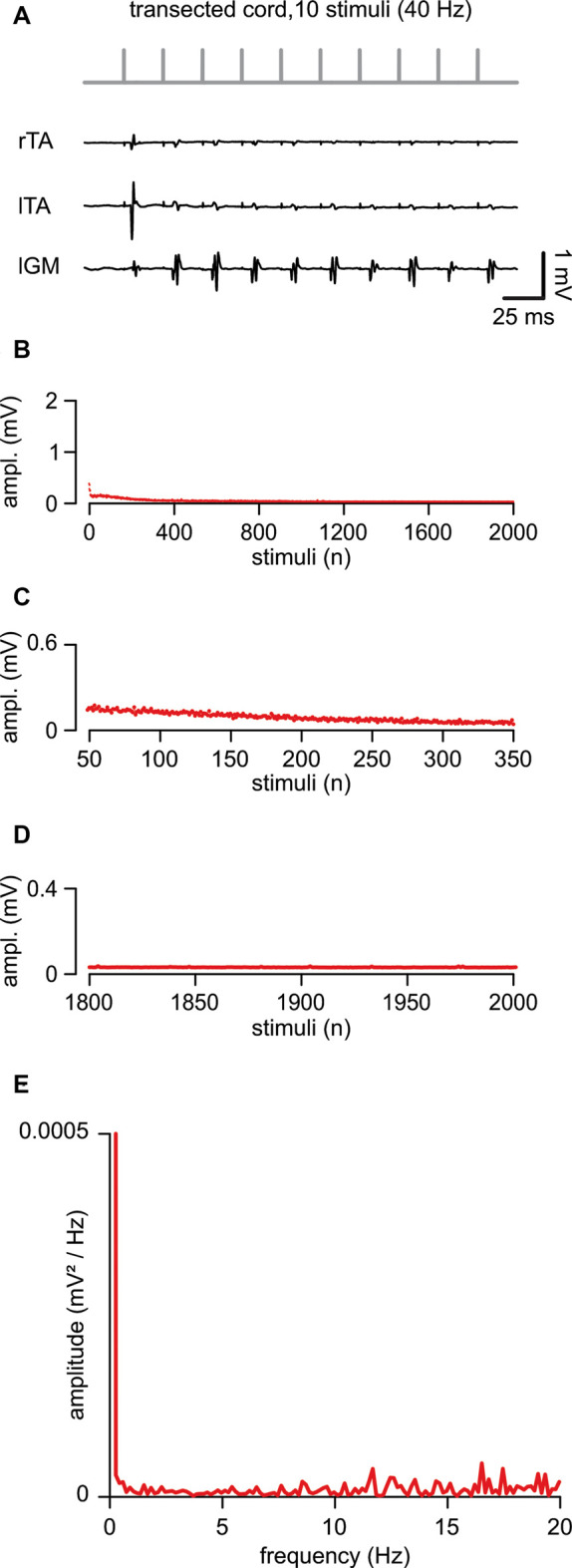
An acute transection perturbs the peak amplitude of repetitive, spinally-induced reflexes. **(A)** After transection of the cord (Th 10/11), a train of 10 stimuli applied to the cord (L5 spinal level) at 40 Hz evokes EMG reflexes from bilateral tibialis anterior (TA) and left gastrocnemius (GM) muscles. **(B)** Mean time course of the amplitude of responses over the entire length of a stimulation protocol (2,000 stimuli at 40 Hz). Data pooled from five animals with a transected spinal cord. **(C)** Magnification of the time-course in **(B)** draws responsesto the first 300 stimuli (from the 50th to the 350th). **(D)** Magnification of the time-course in **(B)** shows the profile of peaks of the last 200 responses(from the 1,800th to the 2,000th). **(E)** The power spectrum of the mean time-course for five animals illustrates the rhythmic components of an oscillatory pattern of modulation of the peak amplitude throughout the entire stimulation protocol.

In five animals, an acute transection of the spinal cord at the low thoracic level did not affect motor responses to single epidural pulses applied below the lesion site (duration = 0.1 ms; intensity = 1.34 ± 0.44 mA). When compared to intact control responses, spinal reflexes elicited by single suprathreshold stimuli, after acute injury, showed unchanged mean latency (5.29 ± 1.04 ms; *P* = 0.738, *t*-test, *t* = 0.343; *n* = 8, 5) and mean amplitude (0.43 ± 0.26 mV; *P* = 0.549; *t*-test, *t* = 0.618; *n* = 8, 5). In Figure 4A, in a representative animal, the first ten reflexes elicited by a train at 40 Hz were derived from three muscles. Although spinal reflexes to the first pulse seemed unaffected by transection, they showed more frequent failures to the following nine stimuli (Figure 4A). To further explore responses to repetitive stimulation after an acute spinal transection, consecutive motor responses were induced by delivering a train of pulses (2,000 stimuli, 40 Hz). In Figure 4B, the average time-course of peak amplitudes was traced from five experiments. Compared to intact animals, consecutive reflexes significantly faded away faster during the first 300 stimuli (7.5 s of stimulation; Figures 4B,C; 44.63 ± 22.57% to control; *P* = 0.005, *t*-test, *t* = 3.810; *n* = 5) and were completely abolished after approximately the 800th pulse (~25 s of stimulation) under continuous stimulation at the same intensity (Figures 4B,D). Collectively, the mean cumulative amplitude of all 2,000 motor reflexes evoked by the stimulation protocol in injured spinal cords is significantly lower concerning intact control spinal cords (51% of intact controls; *P* = 0.045; Mann–Whitney rank-sum test; *n* = 8, 5).

The power spectrum of the mean time-course was largely suppressed (RMS = 0.077 ± 0.02; *n* = 5; Figure 4E), but still displayed a single principal component of modulation that is equal to intact controls (1.41 ± 0.33 Hz, *n* = 5), albeit of negligible amplitude (0.00002 ± 0.0002 mV/Hz^2^; *n* = 5).

In summary, in contrast to intact animals, after an acute transection, the magnitude of motor response modulation to repeated ESS was abolished within the first few seconds (typically, less than 10 s) from stimulation onset, with the amplitude scarcely modulated by the endogenous rhythmic pattern.

### The Selective A1 Competitive Antagonist, DPCPX Did Not Affect the Motor Output Induced by Electrical Stimulation Immediately Following Spinal Transection

In the current study, we reported that DPCPX augmented the amplitude of repetitive responses in intact animals. Moreover, we indicated that, after an acute spinal transection, the motor output elicited by a train of pulses was dramatically reduced. To verify whether the selective antagonism of A1 receptors could rescue motor reflexes immediately following spinal transection, increasing concentrations of DPCPX were applied to the lumbosacral cord below the level of transection. However, no differences were revealed in the cumulative amplitude of reflexes, even at higher DPCPX concentrations (50–100 μM; *P* = 0.953; Kruskal–Wallis one-way ANOVA on Ranks; *n* = 5), following transection when compared to baseline responses.

## Discussion

Despite the promise of ESS in subjects with SCI (Angeli et al., 2018; Gill et al., 2018; Wagner et al., 2018), variability in patient responses and sub-restorative levels of improvement indicate the need for the development of co-therapies to improve the clinical efficacy of ESS therapy. Combining spinal electrical neuromodulation with pharmacological approaches presents an appealing mechanism to augment the effectiveness of neurorehabilitation. Here, we demonstrate that selective pharmacological blockage of A1 subtype adenosine receptors during spinal electrical stimulation facilitates sensorimotor networks in the intact spinal cord. A1 inhibition results in an increased magnitude of an endogenous pattern that modulated spinally-evoked motor potentials and sustained duration of ESS-induced activity.

### Motor Output to Epidural Stimulation Is Affected by an Endogenous Pattern of Modulation

In the clinic, repetitive ESS with a train of electrical pulses has been applied at different frequencies to elicit distinct types of motor recovery in select groups of young adults with chronic and complete SCI (Barolat et al., 1986; Jilge et al., 2004; Minassian et al., 2007; Harkema et al., 2011; Grahn et al., 2017). This pattern of stimulation elicits a series of spinally-evoked motor potentials, which are, in nature, similar to spinal reflexes from hindlimb muscles (Sayenko et al., 2014; Hofstoetter et al., 2018). However, the response to each delivered pulse can be periodically modulated during repetitive spinal stimulation of the same frequency and intensity (Hofstoetter et al., 2015; Sayenko et al., 2019). Similarly, here we demonstrate that the amplitude of spinally-induced reflexes can vary stochastically during repetitive epidural stimulation in fully anesthetized animals. These fluctuations of peak responses have also been observed previously in anesthetized animals that were continuously subjected to pulses train at a subthreshold intensity and lower frequency (i.e., 0.3 Hz) than those used in this study (Taccola et al., 2020a). Based on this evidence, we recently postulated that an endogenous pattern of modulation of the motor output originates from spinal networks (Taccola et al., 2020a). Present data corroborate that an endogenous pattern of modulation of spinally-induced reflexes occurs in response to a stimulation protocol that replicates the characteristics of a standard clinical ESS procedure (40 Hz, suprathreshold intensity). Moreover, power spectral density analysis of the reflex peaks’ time-course shows that repetitive spinally-evoked motor potentials are rhythmically modulated. Indeed, we consistently observed that the main component of the periodic pattern of modulation was more pronounced in amplitude every ~50 stimuli, which is at ~0.8 Hz. A similar endogenous sinusoidal pattern originating from spinal circuits has been reported during rhythmic modulation of the amplitude of motor output in behaving cats (Cuellar et al., 2009).

It remains to be seen whether the optimal frequency of stimulation should be tuned to the intrinsic physiologic pattern of activity to maximize the effectiveness of neuromodulation.

In particular, it is yet to be explored whether spinal stimulation implemented by delivering trains of stimuli within the range shown to be physiologically effective and containing the principal components of the intrinsic physiological patterns of modulation (Gerasimenko et al., 2005; Lavrov et al., 2006), can modulate spinal networks to a more receptive state. Alternatively, it remains to be explored to what extent do the failures of stimulation to induce a response to contribute to the summation of the membrane potential of spinal neurons, thereby affecting spinal neuron thresholds at periodic higher frequencies.

### Neurochemical Response to ESS

During training sessions in the presence of stimulation in humans, the motor output cannot be sustained at the same level and has been noticed to decline with the duration of the training and stimulation (Rejc et al., 2017; Gill et al., 2018; Sayenko et al., 2019). Multiple factors can contribute to this decline of a patient’s performance, including cardiovascular and muscle fatigue which are proportionally dependent on the duration and amount of motor activity. However, this decline of performance must also be considered in the context of neurochemical responses to ESS. It is critical to note that spinal stimulation drives multiple responses. For example, spinal stimulation simultaneously facilitates the release of many neurotransmitters and neuromodulators because of the different synapses encountered by the electrical field in the spinal cord (Taccola et al., 2018). This increase of neurotransmitters and neuromodulators acts at the synaptic milieu of multiple network sites, including their release from the same Ia afferents that were involved during a continuous repetitive stimulation (Kuno, 1964; Hofstoetter et al., 2019). Through this release of multiple neurotransmitters and neuromodulators, ESS might mimic the physiological changes in the chemical environment of the spinal cord during rhythmic activities (e.g., locomotion) or lead to synaptic “fatigue” and induction of receptor sensitization (Taccola et al., 2018). In the current study, the amplitude of muscle responses to 40 Hz stimulation of suprathreshold intensity decreased until full response abolishment occurred after less than 45 s. This fact is reminiscent of the spontaneously decaying locomotor-like oscillations evoked by electrical stimulation in the isolated spinal cord, even during the continuous delivery of pulses (Marchetti et al., 2001b). This failure has been ascribed to presynaptic mechanisms associated with a diminished release of glutamate during prolonged stimulation. Besides, at the postsynaptic level, a membrane shunt determined by a local increase of potassium concentrations in the extracellular milieu should also be considered (Marchetti et al., 2001a). Further, the release of inhibitory neurotransmitters triggered by continuous stimulation can contribute to the decay of responses (Dale and Gilday, 1996). The present results indicate that, although a strong suppression of spinally-induced reflexes occurs after ~45 s of the stimulation protocol, a brief 5-min rest was sufficient to promote the reemergence of suprathreshold motor responses between repetitions. This prompt recovery of the evoked motor output helps to explain the efficacy of burst spinal cord stimulation over the 40 Hz “gold standard” used for spinal stimulation protocols in experimental animal models (Taccola, 2011; Meuwissen et al., 2019; Taccola et al., 2020a). Indeed, when trains of stimuli (e.g. 60 s) were repetitively applied over a long time frame (e.g., 45 min) to spinal cords isolated from neonatal rats, 2-min pauses appeared necessary to equally activate spinal circuits for locomotion (Dingu et al., 2016). The self-limiting properties of the spinal circuits that process repetitive pulses indicate the need to include periodic pauses during continuous ESS to maximize the expression of motor responses.

The facilitatory effect of the A1 antagonist on the amplitude of motor output illustrated in the current study is explained by two tentative mechanisms. First, DPCPX selectively blocks inhibitory presynaptic receptors on primary afferents to maximize input delivery. Second, the antagonism of postsynaptic A1 receptors depressing the activity of spinal networks (Witts et al., 2015) might increase the excitability of the spinal cord, in turn, maximizing the magnitude of motor output.

### An Acute Spinal Transection Depresses the Response of Repetitive Spinal Cord Stimulation

In the present study, although DPCPX largely increased the yield of repetitive stimulation in intact cords, after a spinal transection the selective antagonism of A1 receptors appeared to be ineffective. This can be in line with the contrasting and paradoxical effects of adenosine agonists and antagonists reported in various neurodegenerative conditions (Stone et al., 2009). For instance, a previous study showed a massive release of adenosine right after a spinal lesion (McAdoo et al., 2000), and adenosine is putatively able to compete with DPCPX at A1 receptor sites. In our study, an acute spinal cord transection affected the amplitude of reflexes evoked by repetitive stimulation. Moreover, the endogenous pattern of modulation was largely silenced immediately after spinal transection, without the periodic appearance of higher peaks that occur in intact cords under the stimulation protocol. Following an acute SCI, a period of several hours to a few weeks has been reported both in humans and in animals (Lavrov et al., 2008), wherein electrophysiological signals, such as spinal reflexes, are depressed (Ditunno et al., 2004). The mechanisms of this reduced neural activity remain poorly understood. At the same time, although the existence of an acute period of spinal shock in rats has been reported after severe spinal contusions (Taccola et al., 2020b) it is rarely the case immediately after complete surgical transection of the cord conducted under full anesthesia (Coskun et al., 2010). Our study demonstrates that spinal reflexes in response to single epidural pulses remain unaffected by spinal transection, similar to what has been reported in awake rats (Lavrov et al., 2006). However, the responses to repetitive stimuli were reduced. We can speculate that the transection of descending and propriospinal projections reduces the extent of lumbar spinal circuits by interrupting reverberating polysynaptic pathways possibly involved in the modulation of repetitive responses. Still, whether similar effects are also present in chronic injuries needs to be demonstrated.

## Conclusions

In the current study, we further explore how the amplitude of motor responses evoked by repetitive pulses applied to a rodent spinal cord is modulated by the endogenous spinal rhythm (Taccola et al., 2020b). This endogenous pattern of modulation is different from the central pattern generator for locomotion (Dimitrijevic et al., 1998) since it has not been able, *per se*, to generate any coordinated motor activity from hindlimb muscles. Rather, it can be speculated that the endogenous pattern of modulation represents an intrinsic and rhythmic tone able to set the subthreshold excitability of propriospinal circuits (Taccola et al., 2020a). At the cellular level, it should be viewed as synchronized spontaneous oscillations occurring in the resting potentials of circuit elements that do not reach the threshold for triggering an action potential, but which can still summate incoming external input. Synchronized subthreshold changes in membrane potentials spontaneously occur in microcircuits *in vitro* (Lampl and Yarom, 1993; Lefler et al., 2020). It seems imperative now to explore if a similar pattern of modulation can be found in the human spinal cord. This information will help better define the whole duration of each ESS session and tune the frequency of neuromodulating paradigms, thereby increasing the extent of motor recovery. Although in the current study, DPCPX was unable to facilitate neuromuscular response during spinal cord stimulation in acute spinal cord transected rats, this is likely due to temporary ionic disruption of spinal networks. Future research should include additional preclinical studies in chronically injured animals to elucidate a potential role of the selective pharmacological A1 antagonism in maximizing the recovery of motor output during ESS and compare the effects with previously used pharmacological neuromodulation agents within the same protocol. Also, spinal electrical neuromodulation has recently received great attention as an innovative rehabilitation tool for several neuromotor conditions that do not affect the spinal cord directly, such as multiple sclerosis (Illis et al., 1980), Parkinson’s disease (Santana et al., 2014; de Andrade et al., 2016; Samotus et al., 2018; de Souza et al., 2018), and cerebellar ataxia (Solopova et al., 2017). For these pathologies, A1 antagonists are a potential new avenue for restoring sensorimotor functions in the presence of spinal stimulation. At the same time, more detailed and intensive preclinical studies are needed to investigate dosage, optimize methods of delivery, and potential systemic effects and precautions, before the synergistic application of A1 antagonists and spinal stimulation is translated to clinic.

## Data Availability Statement

The raw data supporting the conclusions of this article will be made available by the authors, without undue reservation.

## Ethics Statement

The animal study was reviewed and approved by Institutional Animal Care and Use Committee at Houston Methodist Research Institute.

## Author Contributions

GT conceptualized the study. GT, DS, PJH, BHS, and MKH performed experiments. GT, RA, and DS analyzed data. GT prepared figures. GT and DS drafted the manuscript. All authors revised the manuscript and approved the final version. All authors agree to be accountable for all aspects of the work in ensuring that questions related to the accuracy or integrity of any part of the work are appropriately investigated and resolved.

## Conflict of Interest

The authors declare that the research was conducted in the absence of any commercial or financial relationships that could be construed as a potential conflict of interest.

## Abbreviations

ATP: adenosine triphosphate
ANOVA: analysis of variance
CNS: central nervous system
EMG: electromyogram
ESS: epidural spinal stimulation
GM: gastrocnemius
i.p.: intraperitoneal
l: left
L: lumbar
SCI: spinal cord injury
SD: standard deviation
TA: tibialis anterior
Th: thoracic
DPCPX: 8-cyclopentyl-1,3-dipropylxanthine
DMPX: 3,7-dimethyl-1-propargylxanthine.

## References

[B1] Acton D. Miles G. B. (2015). Stimulation of glia reveals modulation of mammalian spinal motor networks by adenosine. PLoS One 10:e0134488. 10.1371/journal.pone.0134488 26252389 PMC4529192

[B2] Angeli C. A. Boakye M. Morton R. A. Vogt J. Benton K. Chen Y.. (2018). Recovery of over-ground walking after chronic motor complete spinal cord injury. N. Engl. J. Med. 379, 1244–1250. 10.1056/NEJMoa1803588 30247091

[B3] Angeli C. A. Edgerton V. R. Gerasimenko Y. P. Harkema S. J. (2014). Altering spinal cord excitability enables voluntary movements after chronic complete paralysis in humans. Brain 137, 1394–1409. 10.1093/brain/awu038 24713270 PMC3999714

[B4] Aslan S. C. Legg Ditterline B. E. Park M. C. Angeli C. A. Rejc E. Chen Y.. (2018). Epidural spinal cord stimulation of lumbosacral networks modulates arterial blood pressure in individuals with spinal cord injury-induced cardiovascular deficits. Front. Physiol. 9:565. 10.3389/fphys.2018.00565 29867586 PMC5968099

[B5] Aslan S. C. Randall D. C. Krassioukov A. V. Phillips A. Ovechkin A. V. (2016). Respiratory training improves blood pressure regulation in individuals with chronic spinal cord injury. Arch. Phys. Med. Rehabil. 97, 964–973. 10.1016/j.apmr.2015.11.018 26718236 PMC4884550

[B6] Bai H.-H. Liu J.-P. Yang L. Zhao J.-Y. Suo Z.-W. Yang X.. (2017). Adenosine A1 receptor potentiated glycinergic transmission in spinal cord dorsal horn of rats after peripheral inflammation. Neuropharmacology 126, 158–167. 10.1016/j.neuropharm.2017.09.001 28882563

[B7] Barolat G. Myklebust J. B. Wenninger W. (1986). Enhancement of voluntary motor function following spinal cord stimulation—case study. Appl. Neurophysiol. 49, 307–314. 10.1159/000100160 3499118

[B8] Burnstock G. (2007). Physiology and pathophysiology of purinergic neurotransmission. Physiol. Rev. 87, 659–797. 10.1152/physrev.00043.2006 17429044

[B9] Burnstock G. Wood J. N. (1996). Purinergic receptors: their role in nociception and primary afferent neurotransmission. Curr. Opin. Neurobiol. 6, 526–532. 10.1016/s0959-4388(96)80060-2 8794102

[B10] Caciagli F. Ciccarelli R. Di Iorio P. Ballerini P. Tacconelli L. (1988). Cultures of glial cells release purines under field electrical stimulation: the possible ionic mechanisms. Pharmacol. Res. Commun. 20, 935–947. 10.1016/s0031-6989(88)80122-x 3266531

[B11] Coskun C. Avci B. Ocak N. Yalcin M. Dirican M. Savci V. (2010). Effect of repeatedly given CDP-choline on cardiovascular and tissue injury in spinal shock conditions: investigation of the acute phase. J. Pharm. Pharmacol. 62, 497–506. 10.1211/jpp.62.04.0013 20604840

[B12] Courtine G. Gerasimenko Y. van den Brand R. Yew A. Musienko P. Zhong H.. (2009). Transformation of nonfunctional spinal circuits into functional states after the loss of brain input. Nat. Neurosci. 12, 1333–1342. 10.1038/nn.2401 19767747 PMC2828944

[B13] Cuellar C. A. Tapia J. A. Juarez V. Quevedo J. Linares P. Martinez L.. (2009). Propagation of sinusoidal electrical waves along the spinal cord during a fictive motor task. J. Neurosci. 29, 798–810. 10.1523/JNEUROSCI.3408-08.2009 19158305 PMC6665157

[B14] Dale N. Gilday D. (1996). Regulation of rhythmic movements by purinergic neurotransmitters in frog embryos. Nature 383, 259–263. 10.1038/383259a0 8805702

[B15] Darrow D. Balser D. Netoff T. I. Krassioukov A. Phillips A. Parr A.. (2019). Epidural spinal cord stimulation facilitates immediate restoration of dormant motor and autonomic supraspinal pathways after chronic neurologically complete spinal cord injury. J. Neurotrauma 36, 2325–2336. 10.1089/neu.2018.6006 30667299 PMC6648195

[B16] de Andrade E. M. Ghilardi M. G. Cury R. G. Barbosa E. R. Fuentes R. Teixeira M. J.. (2016). Spinal cord stimulation for Parkinson’s disease: a systematic review. Neurosurg. Rev. 39, 27–35. 10.1007/s10143-015-0651-1 26219854

[B17] de Leon R. D. Tamaki H. Hodgson J. A. Roy R. R. Edgerton V. R. (1999). Hindlimb locomotor and postural training modulates glycinergic inhibition in the spinal cord of the adult spinal cat. J. Neurophysiol. 82, 359–369. 10.1152/jn.1999.82.1.359 10400964

[B18] de Souza C. P. Dos Santos M. G. G. Hamani C. Fonoff E. T. (2018). Spinal cord stimulation for gait dysfunction in Parkinson’s disease: essential questions to discuss. Mov. Disord. 33, 1828–1829. 10.1002/mds.27508 30485907

[B19] Deumens R. Mazzone G. Taccola G. (2013). Early spread of hyperexcitability to caudal dorsal horn networks after a chemically-induced lesion of the rat spinal cord *in vitro*. Neuroscience 229, 155–163. 10.1016/j.neuroscience.2012.10.036 23103212

[B20] Dimitrijevic M. R. Gerasimenko Y. Pinter M. M. (1998). Evidence for a spinal central pattern generator in humans. Ann. N Y Acad. Sci. 860, 360–376. 10.1111/j.1749-6632.1998.tb09062.x 9928325

[B21] Dingu N. Deumens R. Taccola G. (2016). Electrical stimulation able to trigger locomotor spinal circuits also induces dorsal horn activity. Neuromodulation 19, 38–46. 10.1111/ner.12354 26449748

[B22] Ditunno J. Little J. Tessler A. Burns A. (2004). Spinal shock revisited: a four-phase model. Spinal Cord 42, 383–395. 10.1038/sj.sc.3101603 15037862

[B23] Edgerton V. R. Courtine G. Gerasimenko Y. P. Lavrov I. Ichiyama R. M. Fong A. J.. (2008). Training locomotor networks. Brain Res. Rev. 57, 241–254. 10.1016/j.brainresrev.2007.09.002 18022244 PMC2288528

[B24] Fisone G. Borgkvist A. Usiello A. (2004). Caffeine as a psychomotor stimulant: mechanism of action. Cell. Mol. Life Sci. 61, 857–872. 10.1007/s00018-003-3269-3 15095008 PMC11138593

[B25] Fong A. J. Roy R. R. Ichiyama R. M. Lavrov I. Courtine G. Gerasimenko Y.. (2009). Recovery of control of posture and locomotion after a spinal cord injury: solutions staring us in the face. Prog. Brain Res. 175, 393–418. 10.1016/S0079-6123(09)17526-X 19660669 PMC2904312

[B26] Freyvert Y. Yong N. A. Morikawa E. Zdunowski S. Sarino M. E. Gerasimenko Y.. (2018). Engaging cervical spinal circuitry with non-invasive spinal stimulation and buspirone to restore hand function in chronic motor complete patients. Sci. Rep. 8:15546. 10.1038/s41598-018-33123-5 30341390 PMC6195617

[B27] Gad P. Gerasimenko Y. Zdunowski S. Turner A. Sayenko D. Lu D. C.. (2017). Weight bearing over-ground stepping in an exoskeleton with non-invasive spinal cord neuromodulation after motor complete paraplegia. Front. Neurosci. 11:333. 10.3389/fnins.2017.00333 28642680 PMC5462970

[B28] Geiger J. LaBella F. Nagy J. (1984). Characterization and localization of adenosine receptors in rat spinal cord. J. Neurosci. 4, 2303–2310. 10.1523/jneurosci.04-09-02303.1984 6090615 PMC6564794

[B29] Gerasimenko Y. P. Lavrov I. A. Bogacheva I. N. Shcherbakova N. A. Kucher V. I. Musienko P. E. (2005). Formation of locomotor patterns in decerebrate cats in conditions of epidural stimulation of the spinal cord. Neurosci. Behav. Physiol. 35, 291–298. 10.1007/s11055-005-0059-4 15875491

[B30] Gerasimenko Y. P. Lu D. C. Modaber M. Zdunowski S. Gad P. Sayenko D. G.. (2015). Noninvasive reactivation of motor descending control after paralysis. J. Neurotrauma 32, 1968–1980. 10.1089/neu.2015.4008 26077679 PMC4677519

[B31] Gill M. L. Grahn P. J. Calvert J. S. Linde M. B. Lavrov I. A. Strommen J. A.. (2018). Neuromodulation of lumbosacral spinal networks enables independent stepping after complete paraplegia. Nat. Med. 24, 1677–1682. 10.1038/s41591-018-0175-7 30250140

[B32] Grahn P. J. Lavrov I. A. Sayenko D. G. Van Straaten M. G. Gill M. L. Strommen J. A.. (2017). Enabling task-specific volitional motor functions *via* spinal cord neuromodulation in a human with paraplegia. Mayo Clin. Proc. 92, 544–554. 10.1016/j.mayocp.2017.02.014 28385196

[B33] Harkema S. Gerasimenko Y. Hodes J. Burdick J. Angeli C. Chen Y.. (2011). Effect of epidural stimulation of the lumbosacral spinal cord on voluntary movement, standing and assisted stepping after motor complete paraplegia: a case study. Lancet 377, 1938–1947. 10.1016/S0140-6736(11)60547-3 21601270 PMC3154251

[B34] Herrity A. N. Williams C. S. Angeli C. A. Harkema S. J. Hubscher C. H. (2018). Lumbosacral spinal cord epidural stimulation improves voiding function after human spinal cord injury. Sci. Rep. 8:8688. 10.1038/s41598-018-26602-2 29875362 PMC5989228

[B35] Hochman S. Garraway S. M. Machacek D. W. Shay B. L. (2001). 5-HT receptors and the neuromodulatory control of spinal cord function. in Motor Neurobiol. Spinal Cord, eds Cope Timothy C. (CRC Press), 47–87. ISBN: 978-0849300066.

[B36] Hofstoetter U. S. Danner S. M. Freundl B. Binder H. Mayr W. Rattay F.. (2015). Periodic modulation of repetitively elicited monosynaptic reflexes of the human lumbosacral spinal cord. J. Neurophysiol. 114, 400–410. 10.1152/jn.00136.2015 25904708 PMC4507962

[B37] Hofstoetter U. S. Freundl B. Binder H. Minassian K. (2018). Common neural structures activated by epidural and transcutaneous lumbar spinal cord stimulation: elicitation of posterior root-muscle reflexes. PLoS One 13:e0192013. 10.1371/journal.pone.0192013 29381748 PMC5790266

[B38] Hofstoetter U. S. Freundl B. Binder H. Minassian K. (2019). Recovery cycles of posterior root-muscle reflexes evoked by transcutaneous spinal cord stimulation and of the H reflex in individuals with intact and injured spinal cord. PLoS One 14:e0227057. 10.1371/journal.pone.0227057 31877192 PMC6932776

[B39] Illis L. S. Sedgwick E. M. Tallis R. C. (1980). Spinal cord stimulation in multiple sclerosis: clinical results. J. Neurol. Neurosurg. Psychiatry 43, 1–14. 10.1136/jnnp.43.1.1 7354351 PMC490455

[B40] Jilge B. Minassian K. Rattay F. Pinter M. M. Gerstenbrand F. Binder H.. (2004). Initiating extension of the lower limbs in subjects with complete spinal cord injury by epidural lumbar cord stimulation. Exp. Brain Res. 154, 308–326. 10.1007/s00221-003-1666-3 14586532

[B41] Kuno M. (1964). Mechanism of facilitation and depression of the excitatory synaptic potential in spinal motoneurones. J. Physiol. 175, 100–112. 10.1113/jphysiol.1964.sp007505 14241151 PMC1357087

[B42] Lampl I. Yarom Y. (1993). Subthreshold oscillations of the membrane potential: a functional synchronizing and timing device. J. Neurophysiol. 70, 2181–2186. 10.1152/jn.1993.70.5.2181 8294979

[B43] Lavrov I. Dy C. J. Fong A. J. Gerasimenko Y. Courtine G. Zhong H.. (2008). Epidural stimulation induced modulation of spinal locomotor networks in adult spinal rats. J. Neurosci. 28, 6022–6029. 10.1523/jneurosci.0080-08.2008 18524907 PMC2904311

[B44] Lavrov I. Gerasimenko Y. P. Ichiyama R. M. Courtine G. Zhong H. Roy R. R.. (2006). Plasticity of spinal cord reflexes after a complete transection in adult rats: relationship to stepping ability. J. Neurophysiol. 96, 1699–1710. 10.1152/jn.00325.2006 16823028

[B45] Layland J. Carrick D. Lee M. Oldroyd K. Berry C. (2014). Adenosine: physiology, pharmacology and clinical applications. JACC Cardiovasc. Interv. 7, 581–591. 10.1016/j.jcin.2014.02.009 24835328

[B46] Lefler Y. Amsalem O. Vrieler N. Segev I. Yarom Y. (2020). Using subthreshold events to characterize the functional architecture of the electrically coupled inferior olive network. Elife 9:e43560. 10.7554/elife.43560 32043972 PMC7012604

[B47] Marchetti C. Beato M. Nistri A. (2001a). Alternating rhythmic activity induced by dorsal root stimulation in the neonatal rat spinal cord *in vitro*. J. Physiol. 530, 105–112. 10.1111/j.1469-7793.2001.0105m.x 11136862 PMC2278398

[B48] Marchetti C. Beato M. Nistri A. (2001b). Evidence for increased extracellular K^+^ as an important mechanism for dorsal root induced alternating rhythmic activity in the neonatal rat spinal cord *in vitro*. Neurosci. Lett. 304, 77–80. 10.1016/s0304-3940(01)01777-3 11335059

[B49] McAdoo D. J. Robak G. Xu G.-Y. Hughes M. G. (2000). Adenosine release upon spinal cord injury. Brain Res. 854, 152–157. 10.1016/s0006-8993(99)02333-1 10784116

[B50] Meuwissen K. P. de Vries L. E. Gu J. W. Zhang T. C. Joosten E. A. (2019). Burst and tonic spinal cord stimulation both activate spinal GABAergic mechanisms to attenuate pain in a rat model of chronic neuropathic pain. Pain Pract. 20, 75–87. 10.1111/papr.12831 31424152 PMC7004135

[B51] Minassian K. Persy I. Rattay F. Pinter M. M. Kern H. Dimitrijevic M. R. (2007). Human lumbar cord circuitries can be activated by extrinsic tonic input to generate locomotor-like activity. Hum. Mov. Sci. 26, 275–295. 10.1016/j.humov.2007.01.005 17343947

[B52] Musienko P. van den Brand R. Marzendorfer O. Roy R. R. Gerasimenko Y. Edgerton V. R.. (2011). Controlling specific locomotor behaviors through multidimensional monoaminergic modulation of spinal circuitries. J. Neurosci. 31, 9264–9278. 10.1523/jneurosci.5796-10.2011 21697376 PMC3422212

[B53] Nantwi K. D. Goshgarian H. G. (2002). Actions of specific adenosine receptor A1 and A2 agonists and antagonists in recovery of phrenic motor output following upper cervical spinal cord injury in adult rats. Clin. Exp. Pharmacol. Physiol. 29, 915–923. 10.1046/j.1440-1681.2002.03750.x 12207572

[B54] Phillips A. A. Krassioukov A. V. (2015). Contemporary cardiovascular concerns after spinal cord injury: mechanisms, maladaptations and management. J. Neurotrauma 32, 1927–1942. 10.1089/neu.2015.3903 25962761

[B55] Reeve A. J. Dickenson A. H. (1995). The roles of spinal adenosine receptors in the control of acute and more persistent nociceptive responses of dorsal horn neurones in the anaesthetized rat. Br. J. Pharmacol. 116, 2221–2228. 10.1111/j.1476-5381.1995.tb15057.x 8564252 PMC1908979

[B56] Rejc E. Angeli C. A. Atkinson D. Harkema S. J. (2017). Motor recovery after activity-based training with spinal cord epidural stimulation in a chronic motor complete paraplegic. Sci. Rep. 7:13476. 10.1038/s41598-017-14003-w 29074997 PMC5658385

[B57] Rossignol S. Frigon A. (2011). Recovery of locomotion after spinal cord injury: some facts and mechanisms. Annu. Rev. Neurosci. 34, 413–440. 10.1146/annurev-neuro-061010-113746 21469957

[B58] Salter M. Henry J. (1987). Evidence that adenosine mediates the depression of spinal dorsal horn neurons induced by peripheral vibration in the cat. Neuroscience 22, 631–650. 10.1016/0306-4522(87)90359-9 3670602

[B59] Samotus O. Parrent A. Jog M. (2018). Spinal cord stimulation therapy for gait dysfunction in advanced Parkinson’s disease patients. Mov. Disord. 33, 783–792. 10.1002/mds.27299 29442369

[B60] Santana M. B. Halje P. Simplício H. Richter U. Freire M. A. M. Petersson P.. (2014). Spinal cord stimulation alleviates motor deficits in a primate model of Parkinson disease. Neuron 84, 716–722. 10.1016/j.neuron.2014.08.061 25447740 PMC4428767

[B61] Sayenko D. G. Angeli C. Harkema S. J. Edgerton V. R. Gerasimenko Y. P. (2014). Neuromodulation of evoked muscle potentials induced by epidural spinal-cord stimulation in paralyzed individuals. J. Neurophysiol. 111, 1088–1099. 10.1152/jn.00489.2013 24335213 PMC3949232

[B62] Sayenko D. G. Rath M. Ferguson A. R. Burdick J. W. Havton L. A. Edgerton V. R.. (2019). Self-assisted standing enabled by non-invasive spinal stimulation after spinal cord injury. J. Neurotrauma 36, 1435–1450. 10.1089/neu.2018.5956 30362876 PMC6482915

[B63] Solopova I. Sukhotina I. Zhvansky D. Ikoeva G. Vissarionov S. Baindurashvili A.. (2017). Effects of spinal cord stimulation on motor functions in children with cerebral palsy. Neurosci. Lett. 639, 192–198. 10.1016/j.neulet.2017.01.003 28063935

[B64] Stone T. W. Ceruti S. Abbracchio M. P. (2009). “Adenosine receptors and neurological disease: neuroprotection and neurodegeneration,” in Adenosine Receptors in Health and Disease, eds Wilson C. Mustafa S. (Berlin: Springer), 535–587. 10.1007/978-3-540-89615-9_17 19639293

[B65] Taccola G. (2011). The locomotor central pattern generator of the rat spinal cord *in vitro* is optimally activated by noisy dorsal root waveforms. J. Neurophysiol. 106, 872–884. 10.1152/jn.00170.2011 21613591

[B66] Taccola G. Gad P. Culaclii S. Ichiyama R. M. Liu W. Edgerton V. R. (2020a). Using EMG to deliver lumbar dynamic electrical stimulation to facilitate cortico-spinal excitability. Brain Stimul. 13, 20–34. 10.1016/j.brs.2019.09.013 31585723

[B67] Taccola G. Gad P. Culaclii S. Wang P.-M. Liu W. Edgerton V. R. (2020b). Acute neuromodulation restores spinally-induced motor responses after severe spinal cord injury. Exp. Neurol. 327:113246. 10.1016/j.expneurol.2020.113246 32057795

[B68] Taccola G. Olivieri D. D’Angelo G. Blackburn P. Secchia L. Ballanyi K. (2012). A1 adenosine receptor modulation of chemically and electrically evoked lumbar locomotor network activity in isolated newborn rat spinal cords. Neuroscience 222, 191–204. 10.1016/j.neuroscience.2012.07.030 22824428

[B69] Taccola G. Sayenko D. Gad P. Gerasimenko Y. Edgerton V. R. (2018). And yet it moves: recovery of volitional control after spinal cord injury. Prog. Neurobiol. 160, 64–81. 10.1016/j.pneurobio.2017.10.004 29102670 PMC5773077

[B70] Tawfik V. L. Chang S.-Y. Hitti F. L. Roberts D. W. Leiter J. C. Jovanovic S.. (2010). Deep brain stimulation results in local glutamate and adenosine release: investigation into the role of astrocytes. Neurosurgery 67, 367–375. 10.1227/01.neu.0000371988.73620.4c 20644423 PMC2919357

[B71] Tresch M. C. Bizzi E. (1999). Responses to spinal microstimulation in the chronically spinalized rat and their relationship to spinal systems activated by low threshold cutaneous stimulation. Exp. Brain Res. 129, 401–416. 10.1007/s002210050908 10591912

[B72] Wagner F. B. Mignardot J. B. Le Goff-Mignardot C. G. Demesmaeker R. Komi S. Capogrosso M.. (2018). Targeted neurotechnology restores walking in humans with spinal cord injury. Nature 563, 65–71. 10.1038/s41586-018-0649-2 30382197

[B73] Witts E. C. Nascimento F. Miles G. B. (2015). Adenosine-mediated modulation of ventral horn interneurons and spinal motoneurons in neonatal mice. J. Neurophysiol. 114, 2305–2315. 10.1152/jn.00574.2014 26311185 PMC4609759

[B74] Witts E. C. Panetta K. M. Miles G. B. (2011). Glial-derived adenosine modulates spinal motor networks in mice. J. Neurophysiol. 107, 1925–1934. 10.1152/jn.00513.2011 22205649 PMC3331664

